# Occult anterior uveal melanomas presenting as extrascleral extension

**DOI:** 10.1136/bjo-2022-321837

**Published:** 2022-09-01

**Authors:** Abhilasha Maheshwari, Paul T Finger, Codrin E Iacob

**Affiliations:** 1 Ocular Tumor, Orbital Disease, and Ophthalmic Radiation Therapy, The New York Eye Cancer Center, New York, NY, USA; 2 New York Eye Cancer Center, New York, New York, USA; 3 Pathology and Laboratory Science, New York Eye and Ear Infirmary of Mount Sinai, New York, New York, USA

**Keywords:** Choroid, Diagnostic tests/Investigation, Neoplasia, Pathology

## Abstract

**Objective:**

To describe the management of patients with occult anterior uveal melanomas presenting with extrascleral extension.

**Methods and analysis:**

Retrospective case series including five patients with small pigmented nodular mass on the episclera. Each lesion was documented by slit-lamp photography and measured with high-frequency ultrasound imaging (ultrasound biomicroscopy). Diagnosis of uveal melanoma was confirmed by biopsy with lamellar sclerectomy. Immediate scleral patch graft repair was performed. Later, each tumour was treated with palladium-103 ophthalmic plaque brachytherapy. The mean plaque diameter was 12 mm (median, 12; range, 10–14). A mean apex prescription dose of 87 Gy (median, 84.5; range, 82.3–99.2) to a tumour depth of 2 mm from the inner sclera delivered over 7 continuous days. The main outcome measures were best-corrected visual acuity, changes in tumour and scleral characteristics and complications.

**Results:**

During each surgery, residual tumour was visualised within an emissary passageway at the deep plane of scleral resection. At a mean of 80 months (median, 57; range, 24–159) follow-up, no patients experienced graft infection, scleromalacia or rejection. Biopsy was required to establish the diagnosis, transillumination failed, and therefore ultrasound measurements were used to determine the plaque size required to treat the relatively occult intraocular component. Despite these challenges, there were no cases of local tumour recurrence, secondary enucleation or metastatic disease. Attributed to cataract surgery, visual acuities improved in three patients and two were stable.

**Conclusion:**

Extrascleral uveal melanoma extension can occur with undetectable, occult intraocular tumours. In these cases, plaque radiation effectively induced local tumour control, preserved vision and prevented metastasis.

**WHAT IS ALREADY KNOWN ON THIS TOPIC**

- Anterior extrascleral melanoma extension has been shown to occur in association with an easily recognisable underlying tumour.

**WHAT THIS STUDY ADDS**

- This study shows it can occur with an occult uveal melanoma, is diagnosed by scleral tumortumour biopsy and is treatable with palladium-103 plaque radiation therapy.

**HOW THIS STUDY MIGHT AFFECT RESEARCH, PRACTICE OR POLICY**

- This knowledge will help physicians with early intervention, thereby, saving the sight, eye and life of patients with occult uveal melanoma presenting with extrascleral extension.

## Introduction

Uveal melanoma is the most common primary intraocular malignancy and represents 5% of all melanomas diagnosed in the USA.^1 2^ Extraocular tumour extension has been noted to occur in up to 5.8% of patients presenting with uveal melanoma.^3–5^ In addition, the literature and staging according to the 8th edition American Joint Committee on Cancer (AJCC) suggests that the presence of extrascleral extension (ESE) ≥5 mm increases the risks of metastasis and death.^6–10^

Extraocular extension can be primary due to tumour growth or secondary to prior surgery, inflammation, laser or radiation damage.^11–13^ However, that said, the sclera is one of the most radiation-resistant tissues in the body.^8 9^ In addition, primary extraocular extension is more commonly associated with larger, AJCC T3 and T4 sized melanomas. They tend to be located overlying the ciliary body, at vortex veins and among the posterior emissary, juxtapapillary circulation.^14^

Most uveal melanomas can be diagnosed based on clinical presentation and imaging.^15^ Therefore, the classic indications for biopsy included atypical tumours, metastatic tumours with no detectable primary cancer and when the patient wishes histopathological confirmation of the clinical diagnosis.^16^ More recently, biopsies have been performed for genetic analysis to establish risk for metastasis.^17^ In this study, a biopsy was necessary for an atypical reason in that small pigmented episcleral nodules of tumour presented without diagnostic clinical evidence of intraocular tumor.

Herein, we describe the diagnosis of and plaque treatment for a clinical case series of small extrascleral uveal melanomas with occult intraocular tumours.

## Patients and methods

This is a retrospective, interventional, non-comparative case series of five patients with uveal melanoma presenting with ESE. All patient records were available for review. Data recorded at initial visit included the patient’s medical history, clinical findings and ancillary tests including ultrasonography (B-scan), ultrasound biomicroscopy (UBM), optical coherence tomography (OCT), photography (slit-lamp, gonioscopy and fundus) and fundus fluorescein angiography, where indicated. In addition, patient’s systemic evaluation, operative records, histopathological results, follow-up period, complications and final visual outcomes were recorded. All cases were performed by a single surgeon (PTF). Main outcomes were defined as best-corrected visual acuity, surgical complications, local control and metastatic disease.

### Biopsy and histopathology

**Figure 1:**
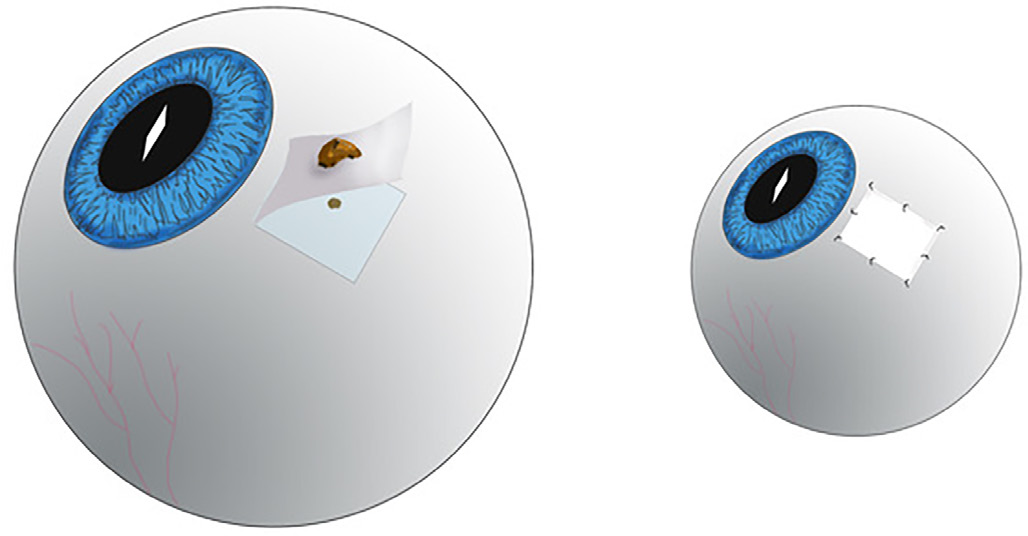
Schematic diagram depicting (left) the half-thickness or deeper scleral flap dissection with round, dark tumour source seen on the sclera beneath the resected tumour. (Right) A scleral patch graft repair was performed using absorbable sutures. (Artist credit: Mr Robert Masini—rmasini@nyee.edu).

All patients underwent tumour and partial thickness scleral diagnostic biopsy. A #57 Bard-Parker blade was used to create a rectangular box, allowing at least 2 mm margins around the edges of the extrascleral tumour nodule (figure 1). Then a half-thickness or deeper scleral flap was dissected as needed to remove scleral and episcleral tumour along with involved conjunctiva. In each case, a round, dark tumour source could be seen on the sclera beneath the resected tumour specimen (figure 1). A scleral patch graft repair was immediately performed using freeze-dried pericardium (Tutoplast, Tutogen Medical, Alachua, Florida, USA). Covering the area of resection, each graft was secured into place with a minimum of four interrupted 6–0 absorbable sutures fastening each corner, then an additional four interrupted sutures in between (figure 1). Lastly, the residual conjunctiva was advanced to the corneal scleral limbus and affixed with 7–0 absorbable sutures as to cover the graft. Subconjunctival bupivacaine and steroid were injected beneath the tenon’s fascia at a position opposite the graft. Patients were discharged with topical antibiotics and steroids.

Histopathological features of all cases were analysed for cell type, presence of melanin, nuclear grade, tumour-infiltrating lymphocytes and necrosis. The patients were informed about the pathology result and the risks associated with untreated residual AJCC T1 intraocular melanomas. Therefore, an initial whole-body positron emission tomography-CT (PET/CT) staging was performed.^18 19^

### Plaque brachytherapy

The 2014 American Brachytherapy Society (ABS) consensus guidelines defined normal ^103^Pd and ^125^I radioactive plaque placement for uveal melanoma as covering the tumour plus a 2–3 mm margin of normal appearing tissue.^20^ In these cases, it was challenging to determine ‘ABS normal’ plaque size. There were no or poor transillumination tumour shadows and almost normative ultrasound measurements for basal dimensions and tumour thickness. In all patients were given episcleral patch graft repair at the time of split-thickness scleral biopsy; the median duration between biopsy and plaque brachytherapy was 6 weeks. Thus, postoperative measurements were complicated by the prior half-thickness sclerectomy biopsy and Tutoplast scleral patch grafts.

For the purposes of this study, we calculated and compared the intraocular radiation dose distribution of these patients as if both ^103^Pd plaque or ^125^I had been used. Additional plaque parameters included: plaque size, type of plaque, apical tumour dose and hours of irradiation. Critical ocular structures were defined as the tumour apex, subjacent sclera, lens, fovea, optic disc and opposite eye wall.^21^

### Follow-up

Ophthalmic examinations were performed every 4–6 months. This included but was not limited to evaluation of the tumour bed documented by slit-lamp and gonio-photography as well as UBM. The posterior segments were evaluated with extended indirect ophthalmoscopy, fundus photography and low-frequency ultrasound imaging. Fluorescein angiography and OCT were performed every 6 months to monitor for radiation vasculopathy. At each visit, test results were compared with the patient’s prior test-examinations for evidence of change. Post-treatment systemic surveillance involved abdominal radiographic imaging every 6 months for the first 5 years and then yearly.^22^

## Results

### Demographic data

Over 36 years of clinical practice, only five patients presented with this unusual form of extrascleral uveal melanoma extension. Their mean age was 52 years (median, 55; range, 33–68). The mean follow-up duration at last visit was 80 months (median, 57; range, 24–159). One patient had a history of breast cancer and one cutaneous basal cell carcinoma. All the tumours were pigmented. Two lesions were temporal and three were nasal (table 1).

**Table 1:** Extrascleral tumour extension by location

Patient	Extrascleral extension location	Clock hour	Distance from limbus (mm)	Preoperative UBM diameter (mm)	Preoperative UBM thickness* (mm)
1	Temporal pars plana	08:00	6	2.9	2.4
2	Nasal pars plana	08:00–09:30	5	4.5	1.3
3	Nasal pars plana	09:00	5.5	4.5	0.9
4	Nasal anterior choroid	15:30	7	4.1	1.4
5	Temporal anterior choroid	09:00	8.5	2	1.6

*For comparison, the normative UBM thickness of the ciliary body is 1.3.^24^

mm, millimetre; UBM, ultrasound biomicroscopy.

### Tumour characteristics

**Figure 2:**
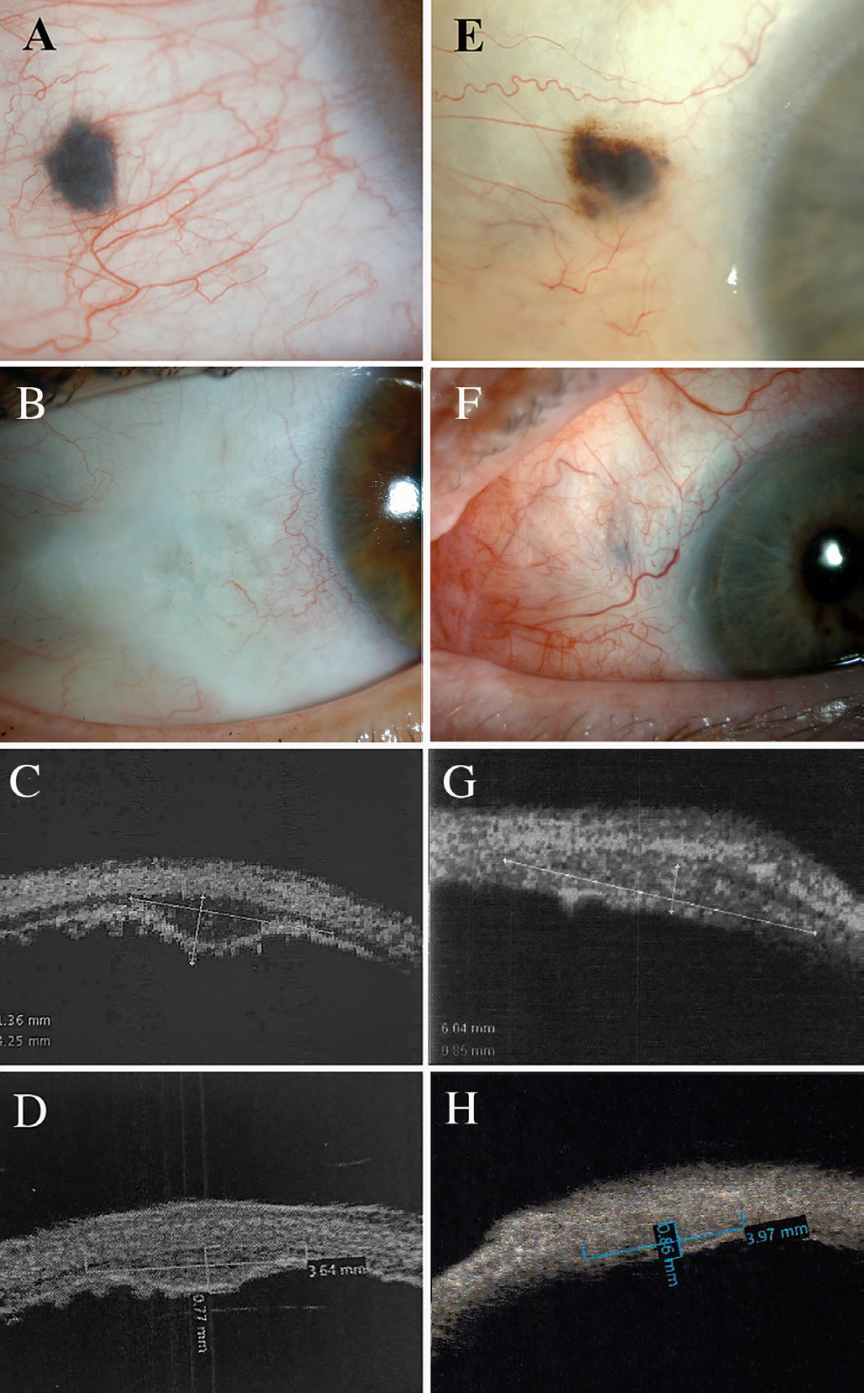
(A) Case #3, a slit-lamp photography shows the small episcleral, melanoma extension prior to biopsy surgery. (B) Case #3, note the cosmetic result 13 years after ^103^Pd plaque brachytherapy. (C) Ultrasound biomicroscopy of case #3 prior to (1.4 mm thickness) and (D) 13 years after brachytherapy (0.8 mm thickness). (E) Slit-lamp photography of case #4 reveals a more discohesive pigmented nodule of episcleral extension before biopsy and (F) 9 years after brachytherapy. (G) Ultrasound biomicroscopy of case #4 before biopsy (0.9 mm thickness) and (H) 9 years after brachytherapy (0.9 mm thickness). In both cases, UBM reveals persistent scleral integrity, increased intratumour reflectivity and excellent local control. UBM, ultrasound biomicroscopy.

Pretreatment tumour characteristics of the ESE are found in table 1. As demonstrated by case #3, note the pigmented round cohesive episcleral melanoma (figure 2A). Thirteen years after palladium-103 plaque brachytherapy, note no evidence of episcleral growth and an excellent cosmetic result (figure 2B). In case #4 the melanoma is relatively discohesive, with subconjunctival pigment dispersion (figure 2E). Although the overlying conjunctiva was sent for biopsy, its status was not commented on by our pathologist nor was it then included within the irradiated zone. In case #4, there was some scleral graft thinning 10 years after brachytherapy (figure 2F).

Of interest, the mean pretreatment largest basal intraocular tumour dimensions as measured by UBM were 3.6 mm (median, 4.1; range, 2–4.5) and the mean pretreatment tumour height was only 1.5 mm (median, 1.4; range, 0.9–2.4). UBM of the tumour for case #3 showed 1.4 mm thickness (figure 2C) which reduced to 0.8 mm (figure 2D), 13 years after brachytherapy. For case #4, UBM showed 0.9 mm thickness (figure 2G) before surgery which remained the same 9 years after brachytherapy (figure 2H). Note that in both cases, the internal acoustic reflectivity increased as was consistent with local control.

According to the 8th edition AJCC staging system for uveal melanoma, the category of anatomic extent for all 5 (100%) tumours was T1cN0M0 (stage IIA) in that extraocular extension was ≤5 mm in all patients.^23^ The mean intraocular tumour thickness from inner sclera, measured at last follow-up was 0.4 mm (median, 0.4 mm; range, 0.0–0.7 mm) or 73.4% less than the preoperative value.

### Histopathology

A histopathological diagnosis of uveal melanoma was established in each case. Histopathological evaluation of the melanomas revealed that 40% (n=2/5) contained epithelioid cells and 60% (n=3/5) were mixed cell tumours. The mean tumour depth on the histopathological specimen was 0.6 mm (median, 0.6; range, 0.2–0.9). The mean largest histopathological basal tumour dimension was 2 mm (median, 0.8; range, 0.5–4.2). The channels between the ESE and the uvea were not seen on histopathology. As was thought due to the typically diagonal path of emissary vessels through the sclera.^24^

### Radiation dosimetry

Before resection, the mean uveal melanoma depth (largest chorioretinal thickness) beneath the biopsy site by UBM was 1.5 mm (median, 1.4; range, 0.9–2.4). In consideration of their small size, we used a minimum axial intraocular radiation prescription distance of 2.0 mm (from inner sclera) in all cases. The tumour’s basal dimensions were based on the UBM appearance of the uveal shadows noted on transillumination prior to biopsy. Mean plaque diameter was 12 mm (median, 12; range, 10–14). Due to the presence of pigmented tumour against the pigmented ora serrata, intraoperative tumour transillumination was not positive, hence larger plaque sizes, with resultant larger margins were used (as to cover any occult tumour component).

Then, according to current practice and as reflected in the ABS consensus guidelines, when and extraocular nodule is small, globe sparing plaque irradiation was performed.^4 25^ Preoperative comparative (^103^Pd and ^125^I) intraocular dosimetry was performed using the radionuclides available in our centre.^21^ Table 2 shows the results leading to the use of ^103^Pd in every case.^21 26^

**Table 2:** ^125^I versus ^103^Pd: radiation dose (Gy) delivered to critical ocular structures

Ocular structure			^125^I plaque	^103^Pd plaque	Percent reduction using ^103^Pd (%)
Sclera			156.7	155.8	−0.60
Lens			30.6	28.6	−7.0
Fovea			3.9	1.9	−52.0
Optic disc			4.3	2.2	−51.20
Apex			87	87	Normalised at 0
Actual ^103^Pd radiation dose (in Gy) delivered to critical ocular structures					
Patient No.	Sclera	Lens	Fovea	Optic disc	Apex
1	165.1	50.7	2	2.3	84.1
2	130.2	23.9	1.5	1.4	85.0
3	174.4	10.6	2.9	3.7	82.3
4	173	23.5	1.6	1.6	99.2
5	136.4	34.4	1.7	1.9	84.5
Mean	155.8	28.6	1.9	2.2	87.0

Doses, means for series visual acuity; 1 Gray, 100 cGy; ^125^I, iodine-125; ^103^Pd, palladium-103.

A mean ^103^Pd tumour dose of 87 Gy (median, 84.5; range, 82.3–99.2) was delivered. The mean dose to sclera was 155.8 Gy (median, 165.1; range, 130.2–74.4). The mean radiation doses to lens, optic nerve and fovea were 28.6 Gy (median, 23.9; range, 10.6–50.7), 2.2 Gy (median, 1.9; range, 1.4–3.7) and 1.9 Gy (median, 1.7; range, 1.5–2.9), respectively (table 2). All treatments were continuously delivered over 7 days.

### Visual acuity

Mean pretreatment visual acuity was 20/20 (median, 20/20; range, 20/16–20/32). Myopia was present in 80% (n=4/5) of patients. The post-treatment vision improved to a mean of 20/16 (median, 20/20; range, 20/12.5–20/25) at a mean follow-up of 80 months attributed to three patients having interim cataract surgery during the follow-up period.

### Complications and cosmesis

All patients were phakic at the time of treatment. Three (60%) developed radiation-induced cataract and underwent cataract surgery.^27^ No patient developed radiation maculopathy, optic neuropathy or any observed retinopathy.^28 29^ There was no episcleral graft failure nor scleral necrosis. All patients have been subjectively pleased with their cosmetic results (figure 2).

### Tumour resolution and metastasis

In this series, local tumour control (no recurrence) was achieved in 100% patients. Eye retention was seen in all patients. No patient developed metastasis at the last follow-up.

## Discussion

This series analysed the results of five cases of ESE with occult subjacent uveal melanomas. Biopsy was required to establish the diagnosis. Scleral grafts were placed for tectonic ocular stability, to prevent treatment-induced scleral thinning and for cosmesis. All patients were at risk for scleral thinning as biopsy involved a lamellar scleral dissection followed by episcleral plaque radiation therapy. Treatment resulted in vision retention, local cancer control and lack of metastatic disease. Cataract formation and vision retention were related to the anterior ocular locations of the tumours and thus the radiation source. This resulted in high mean doses to the lens (28.6 Gy) and low doses to the macular fovea (1.9 Gy) as well as the optic nerve (2.2 Gy). We attribute the high rates of local control and lack of metastases to the small tumour sizes (cT1 category by AJCC staging) as well as the use of relatively large treatment margins.

There exist multiple reports of management of small uveal melanomas with ESE (table 3).^30–32^ Bellmann *et al* used ^125^I for 19 patients with uveal melanoma with ESE, where tumour recurrence rates and survival rates were not adversely affected.^4^ Gray *et al* also used ^125^I in a single patient resulting in local control for 2.5 years.^32^ Augsburger *et al* used plaque brachytherapy in eight patients with extrascleral tumour thicknesses varying from 1 to 10 mm. During 10 years follow-up, they noted one local recurrence and three patients with metastasis.^30^ Muen and Damato used ^106^Ru in a single patient with ciliary body melanoma and ESE who showed no local recurrence at 30 months after treatment.^31^ All four small studies provide additional evidence that if the extraocular tumour small and anterior, a globe-sparing irradiation can be performed with local control, eye and vision retention.^4 30–32^

**Table 3:** Plaque brachytherapy for small uveal melanomas with extrascleral extension

Author	Plaque isotope used	No. of patients	Tumour height (mm)	Local recurrence (%)	Follow-up period (months)
Bellmann *et al* 4	^125^I	19	N/A	0	38
Augsburger *et al* 30	^125^I, ^60^Co, ^106^Ru	8	1–10	12.5	120
Muen and Damato^31^	^106^Ru	1	2.0	0	30
Gray *et al* 32	^125^I	1	2.0	0	30
Our study	^103^Pd	5	1.5	0	80

125I = iodine-125, 60Co = cobalt-60, 106Ru = ruthenium-106, 103Pd - palladium-103

*I don’t see any

%, percent; ^60^Co, cobalt-60; ^125^I, iodine-125; mm, millimeters; N/A, not available; No., Number; ^103^Pd, palladium-103; ^106^Ru, ruthenium-106.

In contrast to those studies, our Medline and PubMed searches using the terms: extrascleral, uveal melanoma, diagnostic biopsy and brachytherapy, revealed no cases of epibulbar ESE with occult subjacent uveal melanomas and thus no consensus on their treatment. Contrary to reports that suggest ESE is usually present with the presence of a large uveal melanoma, our series demonstrated that a small, and thus benign looking pigmented epibulbar tumour could be a potentially life-threatening condition.^30^

Uveal melanomas are known to invade sclera through different intrascleral routes.^14 33–35^ Sambuelli *et al* suggested that small tumours with extraocular extension may have originated from melanocytes which are present in the natural emissary channels along arteries, veins and nerves.^24^ Our findings of rounded, dot-like channels at the base of our scleral resections neither prove or disprove Sambuelli’s thesis.

However, the lack of metastatic disease at a mean follow-up of 80 months in our series supports that tumour size (AJCC T-size) at presentation is a significant factor for the prediction of metastasis.^23^ In this series, all tumours were stage IIB (T1N0M0 without ciliary body involvement but with ESE) which have been associated with 89%, 80% and 69% survival at 5 years, 10 years and 15 years, respectively.^5^ Increasing size of tumour and ESE decrease the 5-year survival from 96% to 26% as the stage progresses from AJCC stage I to IIIC.^5^ More recently genetic analysis has been done to establish risk for metastasis.^17^ It is important to note that some of the patients in our study were biopsied prior to the era of genetic analysis. The others were offered, but declined after discussion of the risks, benefits and cost.

The tumour’s basal dimensions were based on the UBM and subjective appearance of the uveal shadows. In this series, UBM was required to make the diagnosis due to the small ‘occult’ intraocular components. Tumour thickness could only be determined by UBM. Both outpatient clinical and intraoperative transillumination were unreliable in these cases, mostly due to the presence of pigmented tumour that lacks contrast with the pigmented ciliary body band. Therefore, relatively large plaque sizes were used so as to cover any missing occult tumour component.

Presence of myopia in 80% patients, in this series, suggests that there could be a component of scleral thinning induced by myopia associated with ESE. However, as the literature reveals no such correlation, this finding might require further research.

The mean radiation dose to lens was 28.6 Gy, which is similar to the dose causing cataract in our prior ^103^Pd plaque study.^27^ Thus it was not surprising that cataract surgery was performed for 60% of our patients.^27 29^ In contrast, the doses to fovea and optic nerve were almost negligible, leading to no radiation retinopathy or optic neuropathy and excellent long-term visual acuity.^28 29^

The limitations of this study are its retrospective nature, small sample size, variable follow-up and lack of control group. However, this observational case series offers unique information including our surgical approach in consideration of variable and somewhat uncertain basal tumour diameters.

In summary, herein is described a case series of small uveal melanomas presenting with ESE, treated by excisional biopsy combined with scleral grafting and staged postoperative ^103^Pd plaque radiation therapy. Our patient’s clinical courses were found to be uncomplicated in that scleral grafting was performed at the time of biopsy, and subsequent ^103^Pd brachytherapy controlled the occult uveal melanomas.

### Methods of literature search

A search of the MEDLINE database was conducted using the keywords extrascleral, uveal melanoma, diagnostic biopsy and brachytherapy. Additional references were culled from the bibliographies of these references. These references were evaluated for their pertinence to the topic, with special consideration given to papers that detailed clinical outcomes of extrascleral tumours.

## Data Availability

All data relevant to the study are included in the article or uploaded as supplementary information.
